# SIRT6 Depletion Suppresses Tumor Growth by Promoting Cellular Senescence Induced by DNA Damage in HCC

**DOI:** 10.1371/journal.pone.0165835

**Published:** 2016-11-08

**Authors:** Namgyu Lee, Hye Guk Ryu, Jung-Hee Kwon, Dae-Kyum Kim, Sae Rom Kim, Hee Jung Wang, Kyong-Tai Kim, Kwan Yong Choi

**Affiliations:** 1 Department of Life Sciences, Pohang University of Science and Technology, Pohang, Gyeongbuk, Korea; 2 Cbs Bioscience Inc., Daejeon, Korea; 3 Donnelly Centre, Departments of Molecular Genetics and Computer Science, University of Toronto, Toronto, Ontario, Canada; 4 Lunenfeld-Tanenbaum Research Institute, Mount Sinai Hospital, Toronto, Ontario, Canada; 5 Department of Surgery, Ajou University School of Medicine, Suwon, Korea; 6 Division of Integrative Biosciences & Biotechnology, Pohang University of Science and Technology, Pohang, Gyeongbuk, Republic of Korea; Institute of Pathology, GERMANY

## Abstract

The role of Sirtuin 6 (SIRT6) as a tumor suppressor or oncogene in liver cancer remains controversial. Thus, we identified the specific role of SIRT6 in the progression of hepatocellular carcinoma (HCC). SIRT6 expression was significantly higher in HCC cell lines and HCC tissues from 138 patients than in an immortalized hepatocyte cell line, THLE-2 and non-tumor tissues, respectively. SIRT6 knockdown by shRNA suppressed the growth of HCC cells and inhibited HCC tumor growth *in vivo*. In addition, SIRT6 silencing significantly prevented the growth of HCC cell lines by inducing cellular senescence in the p16/Rb- and p53/p21-pathway independent manners. Microarray analysis revealed that the expression of genes involved in nucleosome assembly was apparently altered in SIRT6-depleted Hep3B cells. SIRT6 knockdown promoted G2/M phase arrest and downregulation of genes encoding histone variants associated with nucleosome assembly, which could be attributed to DNA damage. Taken together, our findings suggest that SIRT6 acts as a tumor promoter by preventing DNA damage and cellular senescence, indicating that SIRT6 represents a potential therapeutic target for the treatment of HCC.

## Introduction

Hepatocellular carcinoma (HCC) is a major histological subtype, accounting for 85–90% of primary liver cancers[1]. Incidence rates of HCC have remained high in Eastern and South-Eastern Asia and have increased in the United States and Central Europe[2]. Although great efforts have been made to improve the treatment of HCC in the past decade, advanced HCC often has a poor prognosis due to chemotherapeutic resistance and the limited applicability of surgical resection and liver transplantation. Therefore, understanding the precise mechanisms underlying HCC progression and identifying novel therapeutic targets are crucial for the effective treatment of HCC.

Sirtuins are a family of NAD+-dependent deacylases and have been implicated in diverse biological processes and various diseases, including cancer. SIRT6, one of sirtuins, regulates DNA repair, cell metabolism, and transcription by targeting diverse substrates for deacetylation or ADP-ribosylation[3]. SIRT6 is involved in base excision repair or DNA double-strand break (DSB) repair and required for maintaining genomic stability[4]. SIRT6 functions as a histone H3K9Ac deacetylase to repress the transcriptional activities of target genes involved in apoptosis, inflammation, and metabolism[5]. Crucial roles of SIRT6 in DNA repair, metabolism, and inflammation which are associated with cancer have prompted much investigation into the role of SIRT6 in cancer progression. However, the role and mechanistic function of SIRT6 remain elusive in carcinogenesis.

The role of SIRT6 in cancer remains controversial. Contradictory findings as to whether SIRT6 is a tumor repressor or tumor promoter have been reported, even in the same tissue, such as the pancreas[6, 7] or breast[8, 9]. SIRT6 expression is lower in human pancreatic cancer than in healthy tissue[6]. In this context, low levels of SIRT6 upregulated the transcriptional activity of HIF1 and Myc, resulting in accelerated glycolysis and proliferation, respectively[6]. However, another study showed that inhibiting SIRT6 reduced the viability of Capan-1 pancreatic cancer cells treated with gemcitabine, demonstrating the oncogenic potential of SIRT6[10]. In breast cancer, contradictory roles of SIRT6 in drug sensitivity have been reported. The knockdown of SIRT6 increased tumorigenesis and trastuzumab resistance in breast cancer[11] while sensitizing cells to paclitaxel and epirubicin[12].

The reported functions of SIRT6 in liver cancer have also been inconsistent. SIRT6 suppressed the initiation of liver cancer by inhibiting survivin expression in a liver cancer mouse model[13]. SIRT6 expression was lower in cirrhotic and HCC tissues than in normal liver tissues according to a publically available cancer microarray dataset[14]. However, SIRT6 overexpression prevented apoptosis by suppressing BCL2-associated X protein expression, demonstrating the oncogenic potential of SIRT6 in HCC[15]. Furthermore, SIRT6 expression was high in HCC tissues and correlated with poor prognosis[15]. This controversy over the function of SIRT6 in HCC prompted us to investigate the roles and mechanism of SIRT6 in the progression of HCC.

We found that SIRT6 was upregulated more in HCC cell lines and human HCC tissues than in an immortalized hepatocyte cell line and normal tissues, respectively. SIRT6 silencing by shRNA significantly downregulated the growth of HCC cells by inducing cellular senescence in the p16/Rb- and p53/p21-pathway independent manners. SIRT6 knockdown promoted G2/M phase arrest and downregulation of genes encoding histone variants associated with nucleosome assembly, which could be attributed to DNA damage. Our study provides evidence that SIRT6 promotes HCC by preventing cellular senescence induced by DNA damage. These findings suggest that SIRT6 represents a promising therapeutic target for HCC treatment.

## Materials and Methods

### Cell culture

Cell lines, SNU449, SNU475, Huh7 and Hep3B were purchased from the Korean Cell Line Bank (Korea). The cells were cultured at 37°C under 5% CO_2_ in DMEM supplemented with 10% FBS, 100 units/ml of penicillin and 100 μg/ml streptomycin. THLE-2 cell line was purchased from the American Type Culture Collection (USA). THLE-2 cells originated from human primary normal liver cells were plated on culture plates pre-coated with a solution containing 0.01 mg/mL fibronectin, 0.03 mg/mL bovine collagen type I and 0.01 mg/mL bovine serum albumin dissolved in Bronchial Epithelium Basal Medium (BEBM, Lonza). THLE-2 cells were cultured at 37°C under 5% CO_2_ in BEBM supplemented with BEGM SingleQuots (Lonza).

### Cell transfection

To deplete SIRT6 in HCC cells, lentiviral infection was performed as described previously[16]. Briefly, control and SIRT6-depleted cell lines were established utilizing lentivirus particles containing non-target shRNA (SHC016V) and SIRT6-target shRNA (SHCLNV-NM_016539) which were purchased from Sigma (USA). HCC cells were seeded onto plates and infected with lentiviral particles in DMEM containing 8 μg/ml polybrene (Sigma). One day after the incubation, the medium containing lentivirus was changed with the growth medium, and the cells were incubated for another day. Puromycin (Invitrogen) at 2 μg/ml was added to each well to select for cells efficient for SIRT6 depletion. The target sequences of the five shRNA clones are shown in S1 Table.

### Reagent and WST-1 assay

Doxorubicin and etoposide were purchased from Calbiochem (USA) and Sigma (USA), respectively. The effects of those compounds on HCC cell viability were assessed by the WST-1 assay. Cells were incubated with the culture medium containing the WST-1 reagent (Roche) with 1/10 dilution for 48 h after drug treatments. After incubating cells for 1 hr 40 min at 37°C under 5% CO_2_, the absorbance at 450 nm was measured using a microplate reader (Spectrafluor Plus, Tecan).

### Western blotting

Western blotting was performed as described previously[17]. The list of antibodies used for western blotting are shown in S2 Table.

### Colony formation assay

Lentivirus infected cells were plated onto six-well dishes (500 cells per well), and cultured in the growth medium containing 2 μg/ml puromycin for 15 days. The media was replaced with fresh media every three days. The cells were fixed with 4% formaldehyde in PBS and stained with 0.5% crystal violet in 25% methanol for 1 hr. Plates were then washed with PBS to remove excessive dye and photographed with a digital camera. Clonogenicity was quantified by measuring the absorbance of crystal violet at 595 nm after extracting it with 20% acetic acid.

### Soft agar assay

The agar base layer (0.5% agar with the growth medium) was covered in 6-cm plates. A top layer containing 25×10^3^ cells was suspended in 0.35% agarose containing the growth medium and put on top of the base layer. After 5 min, the growth medium was added to prevent agarose gel from drying. Cells were fixed and stained with 4% paraformaldehyde for 20 min and 3.2% crystal violet, respectively, when the size of colonies between two groups was obviously different for Hep3B at the day 16 and for Huh-7 at the day 19.

### Xenograft assay

Five-week-old male nude mice were obtained from Orient Bio (Korea) and raised in a specific pathogen-free room within the animal facilities at POSTECH (Korea). The nude mice were handled according to the “Institutional Animal Care and Use” guidelines of POSTECH. The protocol was approved by the POSTECH Institutional Animal Care and Use Committee of POSTECH. (Permit Number: 2014-02-0011A1). All tumors were obtained after cervical dislocation was performed, and all efforts were made to minimize suffering. Hep3B cells were infected with lentiviral particles containing either control shRNA or SIRT6-shRNA clone 1 or 3. Four days after viral transduction, infected cells (1 x 10^6^) were resuspended in 100 μl serum-free DMEM with the Matrigel basement membrane matrix (BD biosciences) at a 1:1 volume ratio and subcutaneously injected into the right back of the mice. Mice were monitored three times weekly and then daily as their volumes approached 200 mm^3^. General appearance, activity level and body weight were monitored to check the status of health. No mice became severely ill or died at prior to the experimental end point. Tumor sizes were measured every 4 days from 2 weeks after inoculation using a Vernier caliper. Tumor volumes were assessed according to the formula L x S^2^ x 0.52, where L is the longest diameter and S the shortest diameter of the tumor. End point was set to the day when maximum size of control tumor was about 15 mm in diameter. Maximum tumor sizes of each group at the endpoint were 693.1 mm^3^, 302.1 mm^3^ and 376.5 mm^3^The mice were sacrificed by cervical dislocation 38 days after injection and solid tumors were isolated. All tumors were obtained after cervical dislocation was performed, and all efforts were made to minimize suffering.

### Immunofluorescence

To confirm Ki-67 positive cells in the tumor section, solid tumors from mice were placed in formalin for paraffin block preparation. The tumor sections were incubated with mouse anti-Ki-67 antibody (S2 Table) diluted in PBS containing 10% horse serum. Sections were then washed with PBS containing 0.05% Tween-20 and incubated with anti-rabbit Alexa Fluor 594 (Invitrogen) which as diluted in PBS containing 10% horse serum for 1 hr at room temperature in the dark. Sections were then washed with PBS containing 0.05% Tween-20, incubated with Hoechst 33342 (Invitrogen) for 10 min in the dark and mounted with Mounting medium (Dako). To verify γH2AX foci in Hep3B cells, immunocytochemistry was performed as described previously[17]. Following antibodies were used for immunofluorescent staining: anti-γH2AX antibody (S2 Table) and anti-rabbit Alexa Fluor 594 (Invitrogen).

### β-galactosidase senescence assay

After washed in PBS, cells were fixed for 5 min in PBS containing 2% formaldehyde and 0.2% glutaraldehyde. Subsequently cells were incubated with the β-galactosidase staining solution (40 mM citric acid, 5 mM K_4_[Fe(CN)_6_]3H_2_O, 5 mM K_3_[Fe(CN)_6_], 150 mM NaCl, 2 mM MgCl_2_ and 1 mg/ml X-galactosidase) for 5 hrs at 37°C.

### Microarray analysis

The synthesis of target cRNA probes and hybridization were performed using the Agilent’s Low RNA Input Linear Amplification kit (Agilent Technology, USA) according to the manufacturer’s instructions. Briefly, each one μg of total RNA and the T7 promoter primer was mixed and incubated at 65°C for 10 min. The cDNA master mix (5X First strand buffer, 0.1 M DTT, 10 mM dNTP mix, RNase-Out, and MMLV-RT) was prepared and added to the reaction mixer. The samples were incubated at 40°C for 2 hrs and then the dsDNA synthesis was terminated by incubating at 65°C for 15 min. The transcription master mix (4X Transcription buffer, 0.1M DTT, NTP mix, 50% PEG, RNase-Out, Inorganic pyrophosphatase, T7-RNA polymerase, and Cyanine 3/5-CTP) was prepared as directed by the manufacturer. Transcription of dsDNA was performed by adding the transcription master mix to the dsDNA reaction samples and then incubating at 40°C for 2 hrs. Amplified and labeled cRNA was purified by a cRNA Cleanup Module (Agilent Technology) according to the manufacturer’s protocol. Labeled cRNA target was quantified using a spectrophotometer (ND-1000, NanoDrop Technologgy, USA). After checking labeling efficiency, fragmentation of cRNA was performed by adding the 10X blocking agent and the 25X fragmentation buffer, and then incubating at 60°C for 30 min. The fragmented cRNA was resuspended with the 2X hybridization buffer and directly pipetted onto the assembled Agilent’s Human Oligo Microarray (44K). The arrays hybridized at 65°C for 17 hrs using a hybridization oven (Agilent Technology, USA). The hybridized microarrays were washed as directed by the manufacturer.

### Data acquisition and analysis

The hybridized images were scanned using a DNA microarray scanner and quantified with the Feature Extraction Software (Agilent Technology, USA). All data normalization and selection of fold-changed genes were carried out using a software, GeneSpringGX 7.3 (Agilent Technology, USA). The averages of normalized ratios were determined by calculating the average of normalized signal channel intensity divided by the average of normalized control channel intensity. Functional annotation of genes was made according to the procedures of Gene Ontology TM Consortium (http://www.geneontology.org/index.shtml) utilizing the GeneSpringGX 7.3. Gene classification was based on searches done by DAVID (http://david.abcc.ncifcrf.gov/). Data were deposited in the GEO database (http://www.ncbi.nlm.nih.gov/geo/, accession number: GSE75905).

### Cell cycle analysis

To analyze the progression of cell cycle, 1x10^5^ Hep3B cells were seeded onto 6-well plates and transfected with lentivirus containing negative control or SIRT6 shRNA. Four days after incubation, cells were collected after trypsinization, fixed in the fixation solution containing 70% ethanol and 0.5% Tween-20, washed in PBS containing 1% BSA, resuspended in 200 μl PBS containing 100 μg/ml RNase (Sigma) and 50 μg/mL propidium iodide (Sigma), incubated in the dark for 40 min at room temperature, and then analyzed using a flow cytometer (Canto II, BD Biosciences). Acquired data were analyzed using the ModFit LT software (Verity Software House).

### Bromodeoxyuridine (BrdU) incorporation assay

BrdU incorporation assays were conducted in Hep3B cells to estimate DNA replication. Cells, transfected by the respective lentiviruses containing NC and shSIRT6, were incubated in the presence of 10μM BrdU (BD Pharmingen) for 2hours. Cells were then fixed in ethanol, denatured with 2N HCl containing 0.5% Triton X-100, and stained with AlexaFluor647-conjugated anti-BrdU antibody (Invitrogen). Samples were analyzed using both LSR FortessaTM and FlowJo software (Becton-Dickinson, Mississauga, Canada)

### Tissue samples from patients

HCC (n = 115) and surrounding non-tumor hepatic tissues (n = 48) were obtained from 138 HCC patients who had undergone curative resection for primary HCC between 1995 and 2007 at the Ajou Medical Center in Korea. Fresh tumors and surrounding normal tissues were partly snap-frozen in liquid nitrogen immediately after hepatectomy and stored at −80°C. Tissues used in the study were obtained from the patients with written informed consent for the human subjects research; all the potential participants subjected to hepatectomy were given the written form for informed consent to allow them to decide whether or not they want to participate in the study. The informed consent documents signed by the participants at the time of tissue donation have been deposited after the consent procedure and study protocol were approved by the Institutional Review Board of the Ajou Medical Center.

### Real-time RT-PCR

SIRT6 mRNA levels in tumor and non-tumor tissues were determined by real-time RT-PCR as described previously[18]. The mRNA levels of SIRT6 was normalized relative to a set of reference genes including SDHA, HPRT1, HMBS, GAPDH and B2M by subtracting the average expression levels of the five reference genes as an internal control. The mRNA expression levels were calculated as 2^-^Δ^CT^ utilizing ΔC_T_ values (C_T_ of SIRT6 –average C_T_ of reference genes). The primer and probe sequences are shown in S3 Table. mRNA levels of genes regulated by SIRT6 in Hep3B cells were measured similarly by real-time RT-PCR as described previously[16]. The primer sequences are shown in S4 Table. The relative mRNA levels of target genes were determined by the 2^-ΔΔC^_T_ method.

### Statistical analysis

The Box and Whisker plots were used to compare mRNA levels of SIRT6 (2^−ΔCt^ values) between tumors and non-tumors, and differences assessed by Student’s t test. Results from colony forming assays, weights of tumors, immunofluorescence intensities and the WST-1 assay were compared with by bar graphs after analyzed by Student’s t test. *P* values < 0.05 were considered statistically significant, and data marked with a single (*), two (**) or three (***) asterisks for *P* values < 0.05, < 0.01 and < 0.001, respectively. Statistical analyses were performed with SPSS v. 18.0 (IBM) and the open source statistical program R v 3.1.1.

## Results

### SIRT6 knockdown inhibits HCC cell growth *in vitro*

**Fig 1:**
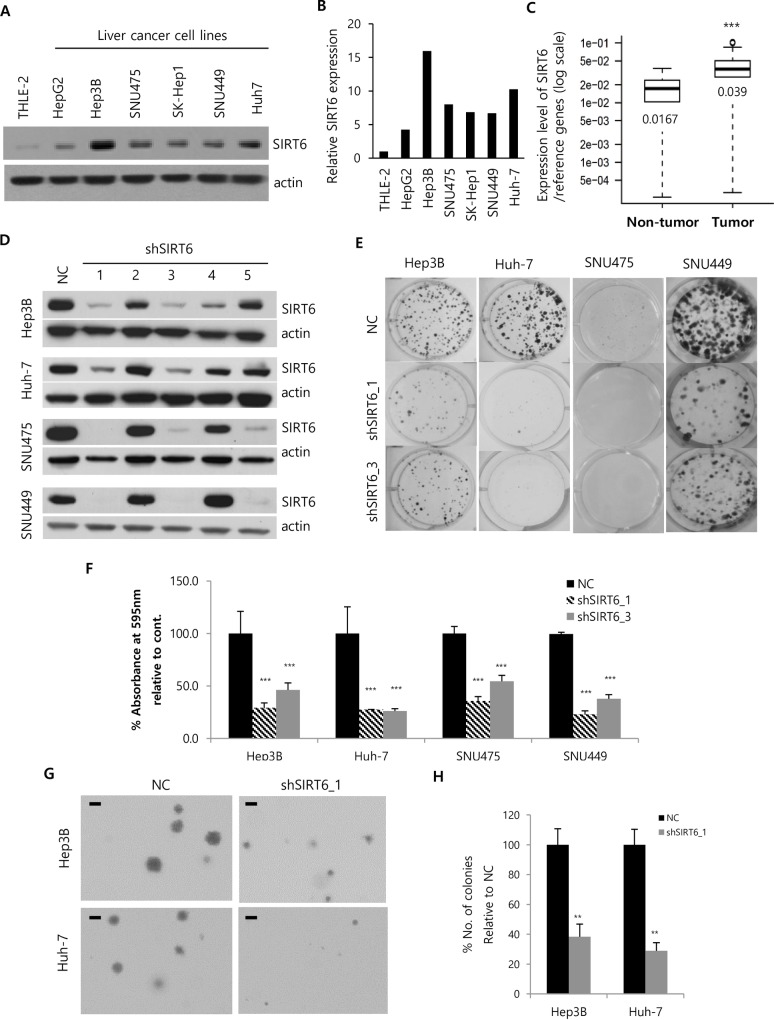
Effects of SIRT6 depletion on HCC cell growth. (A) The expression of SIRT6 in HCC cell lines and normal liver lysate was assessed by western blotting using actin as a loading control. (B) Band intensities were measured by densitometry using Image J. SIRT6 band intensities were normalized to those of actin. (C) Upregulation of SIRT6 mRNA was assessed in HCC tumors relative to normal adjacent tissues by RT-PCR. (D) Hep3B, Huh-7, SNU475, and SNU449 cells were incubated with lentiviral particles containing plasmids with either negative control (NC) or SIRT6 shRNA. Five different lentiviral particles (C1–C5) targeting different sequences were used to delete the SIRT6 gene. HCC cells were harvested 5 days after viral transduction, followed by western blotting to compare the knockdown efficiency of SIRT6. (E) HCC cells transduced by lentiviral particles were seeded into 6-well plates at a density of 500 cells per well. Transduced HCC cells were selected in the growth medium supplemented with 2 μg/ml of puromycin. Cells were fixed and stained with crystal violet after 15 days. Representative images of plates were obtained using a digital camera. (F) The dye was extracted from stained colonies by 20% acetic acid to quantify the colony-forming ability; the extracted dye was quantified by measuring the absorbance at 595 nm with the percentage of the absorbance being calculated relative to that of NC (100%). (G) Hep3B and Huh-7 cells, transduced by the respective lentiviruses containing NC and shSIRT6, were cultured in soft agar with DMEM for 16 and 19 days, respectively. The representative micrographs were obtained using the iSolution Lite program. (H) Colonies larger than the indicated bar on the colony image were counted. The number of colonies (%) was calculated relative to that of NC, which was set to 100%. All data are representative of three independent experiments. (**P<0.05; ***P<0.001).

In order to investigate the role of SIRT6 in liver cancer, the protein levels of SIRT6 were compared among an immortalized hepatocyte cell line, THLE-2, and such six HCC cell lines as Hep3B, HepG2, SNU475, SK-Hep1, SNU449 and Huh-7. SIRT6 levels were higher in liver cancer cells than THLE-2 cells (Fig 1A and 1B). SIRT6 mRNA levels were also measured by RT-PCR in 115 HCC and 48 non-tumor specimens from HCC patients. SIRT6 mRNA levels were significantly elevated in tumor tissues compared with those in non-tumor tissues (Fig 1C; 0.0167 versus 0.039; mean 2^−ΔCT^ values, *P<* 2.2E−16). To investigate the effect of SIRT6 on HCC cell growth, SIRT6 was silenced by shRNA and colony formation assays were then performed. Efficient silencing of SIRT6 was confirmed by western blotting, and lentiviral particles targeting five different sequences showed different knockdown efficiencies (Fig 1D). Clones 1 and 3 showed the most efficient knockdown in HCC cells. SIRT6 knockdown reduced the colony-forming abilities of Hep3B (shSIRT6-1 and -3 by 29.0 ± 4.8% and 46.2 ± 6.6%, respectively), Huh-7 (shSIRT6-1 and -3 by 27.1 ± 0.5% and 26.0 ± 4.2%, respectively), SNU475 (shSIRT6-1 and -3 by 35.3 ± 5.7% and 54.4 ± 4.5%, respectively), and SNU449 (shSIRT6-1 and -3 by 22.7 ± 3.5% and 37.8 ± 4.0%, respectively; *P<0*.001) cells (Fig 1D and 1E). Anchorage-independent growth is another indicator of tumorigenicity. We tested the anchorage-independent growth of SIRT6-deficient cells in soft agar. SIRT6-deficient Hep3B and Huh-7 cells showed significantly lower clonogenicity in soft agar than control cells (Fig 1F and 1G; *P<0*.01).

Recently, SIRT6 mutations were found in several tumor types such as non-small-cell lung cancer, renal clear cell carcinoma, cervical carcinoma, and melanoma[19]. Since naturally occurring tumor-associated mutations in SIRT6 were shown to alter its stability, localization, and/or enzymatic activity[19], we checked SIRT6 status in HCC tissues and cell lines using several public databases such as COSMIC (Catalogue of Somatic Mutations in Cancer, http://cancer.sanger.ac.uk/cosmic) and Cancer Genome Atlas (https://tcga-data.nci.nih.gov/docs/publications/tcga/). Regarding HCC cell lines used for our experiments, mutation status of SK-Hep1 Huh-7, SNU475 and SNU449 could be obtained from COSMIC. Any SIRT6 mutation was not found in these cell lines. In addition, based on databases of COSMIC and Cancer Genome Atlas, only three mutations were found among 1,703 tested samples (mutation rate, 0.17%) and 373 tested samples (mutation rate, 0.8%), respectively. We also confirmed that SIRT6 mutations did not occur in 27 (SIRT6 mutation rate, 0%) and 231 (SIRT6 mutation rate, 0%) HCC samples from two publications published by Japaneses[20] and Korean group[21] that provided whole-genome sequencing of HCC samples.

### Depletion of SIRT6 suppresses tumor growth in a xenograft mouse model

**Fig 2:**
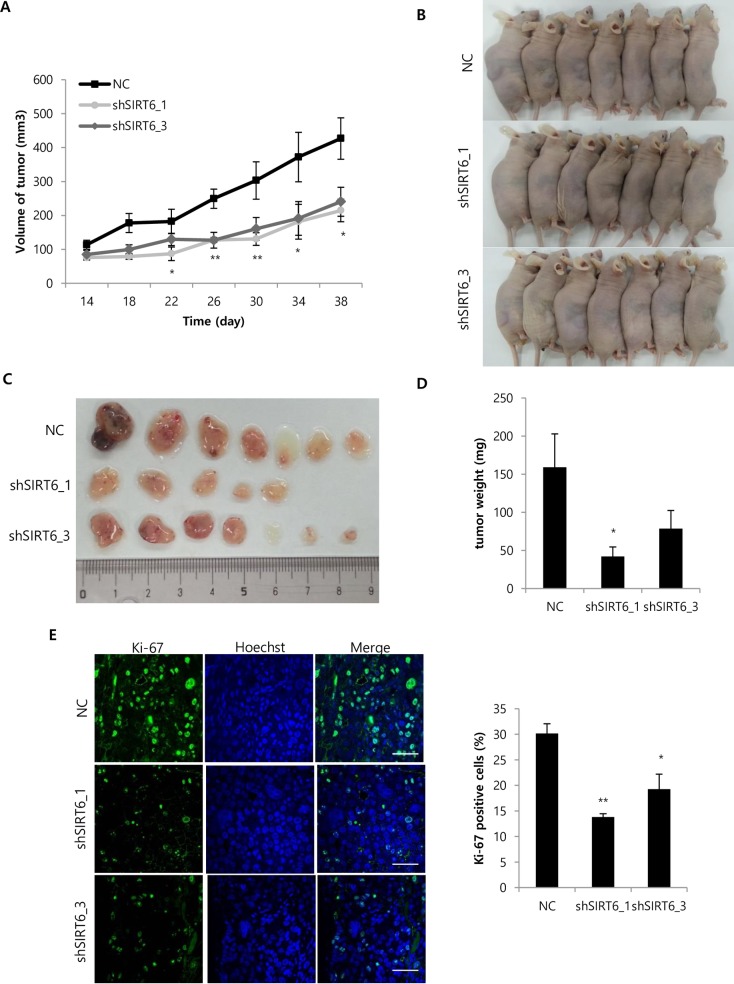
**Effect of SIRT6 depletion on tumor growth in a xenograft mouse model** (A) Hep3B cells were transduced with a lentiviral vector containing NC- or SIRT6-shRNA sequences. Hep3B cells were harvested 4 days after viral transduction, and 1 × 10^6^ cells were subcutaneously injected into mice. Tumor sizes in 21 mice were measured every 4 days after the injection and are shown as means ± SEM. (B) Photographs of mice injected with Hep3B cells were taken after sacrifice. (C) Tumors were obtained from the sacrificed mice, and their sizes were compared. (D) Tumor weights from sacrificed mice are shown as means ± SEM. (E) The expression of Ki-67 was immunohistochemically examined by confocal microscopy, and Ki-67-positive cells were counted in randomly obtained images. The scale bar indicates 50 μM. (*P<0.01, **P<0.05).

To investigate the effect of SIRT6 knockdown on tumor growth *in vivo*, SIRT6-depleted Hep3B cells were inoculated into the flanks of nude mice. Tumor volumes were measured at the indicated time points (Fig 2A). Significant differences in tumor volumes were observed at 22 days post-injection between SIRT6-depleted HCC cells and control HCC cells (*P<0*.05; Fig 2A). Tumor volumes at endpoint for negative control and SIRT6-depleted tumors were 426.7 ± 60.9 mm^3^, 214.7 ± 33.3 mm^3^ and 240.5 ± 42.6 mm^3^, respectively (Fig 2A). After 38 days, mice were sacrificed and solid tumors were removed (Fig 2B and 2C). The weights of SIRT6-depleted tumors (42.0 ± 12.6 mg and 78.6 ± 23.7 mg for shSIRT6-1 and -3, respectively) were lower than those of tumors without SIRT6-deletion (159.1 ± 43.7 mg; Fig 2D). Proliferating cells in the excised tumors were quantified by counting the number of Ki-67-positive cells; fewer Ki-67-positive cells were observed in SIRT6-depleted tumors (shSIRT6-1: 13.8 ± 1.1%, *P<0*.01; shSIRT6-3: 19.2 ± 5.1, *P<0*.05) than in tumors without SIRT6-depletion (30.1 ± 3.3%).

### SIRT6 silencing induces cellular senescence in the p16/Rb and p53/p21-pathway independent manners

**Fig 3:**
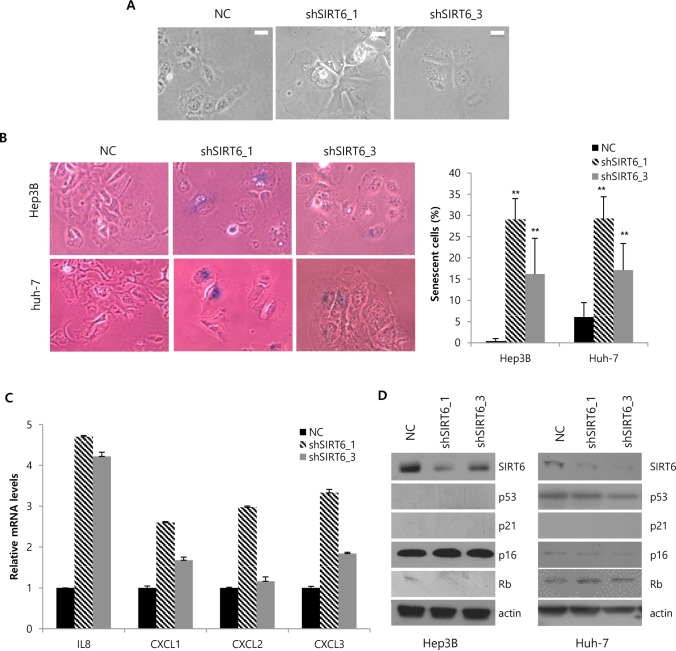
**Induction of cellular senescence by SIRT6 depletion in the p16/Rb and p53/p21- independent manners** (A) Representative micrographs of Hep3B cells 6 days after infection with lentiviruses containing NC or shSIRT6. The scale bar indicates 20 μM. (B) Representative micrographs of SA-ß-gal staining in NC and shSIRT6-depleted Hep3B and Huh-7 cells. The percentage of SA-ß-gal-positive cells was quantified from 6 different areas in the plate. (C) mRNA levels of the selected cytokine genes in control and SIRT6-silenced Hep3B cells are indicated in the histogram. (D) The expression of p16, Rb, p53, and p21 was analyzed by western blotting. Three independent experiments were performed. (**P<0.01).

Next, the nature of growth inhibition induced by SIRT6 deletion was investigated. SIRT6-silenced cells were flattened and larger (Fig 3A) with the characteristic morphology of cellular senescence[17]. ß-gal assays were performed to confirm the induction of cellular senescence by SIRT6 deletion. The percentages of SIRT6-depleted HCC cells positively stained with SA-ß-gal were 29.1 ± 4.9% (shSIRT6-1) and 16.2 ± 8.4% (shSIRT6-3) Hep3B cells and 29.3 ± 5.2% (shSIRT6-1) and 17.1 ± 6.3% (shSIRT6-1) Huh-7 cells, while that of control HCC cells positively stained with SA-ß-gal was less than 1.0% Hep3B cells and 6.1% Huh-7 cells (Fig 3B). Since senescent cells secrete interleukins and cytokines[22], expression levels of several cytokines such as IL8, CXCL1, CXCL2 and CXCL3 were assessed by RT-PCR to validate induction of senescence. SIRT6 silencing increased mRNA levels of IL8, CXCL1, CXCL2 and CXCL3 (Fig 3C). The p53/p21- and p16/Rb-axes are the major senescence-triggering molecular pathways[23], so the expression levels of p53, p21, p16, and Rb were assessed. In Hep3B cells, Rb, p53, and p21 were not detected and p16 expression was not affected by SIRT6 depletion (Fig 3D). In Huh-7 cells, p21 was not detected and p53, p16, and Rb expression was not altered by SIRT6 depletion (Fig 3D).

### Expression of genes involved in nucleosome assembly is significantly altered in SIRT6-depleted Hep3B cells

**Fig 4:**
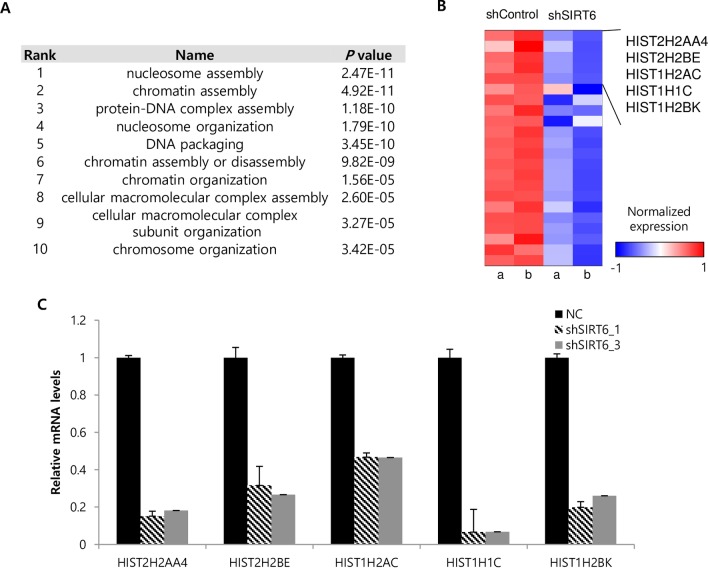
Microarray analysis of SIRT6-depleted Hep3B cells. (A) Microarray data show statistically significant effects of SIRT6 knockdown on gene expression with the indicated gene ontology. (B) The heat map shows the effect of SIRT6 depletion (from two independent Hep3B cell infections designated as “a” and “b”) on genes that function in nucleosome assembly. (C) mRNA levels of the selected nucleosome assembly genes in control and SIRT6-depleted Hep3B cells are indicated in the histogram. All data are representative of two independent experiments.

To identify the mediating effectors contributing to the induction of cellular senescence by SIRT6 depletion, microarray analysis was performed in control and SIRT6-depleted Hep3B cells. Expression of SIRT6 was down-regulated in SIRT6 depleted Hep3B cells by ~50% in our dataset (GEO database, accession number: GSE75905). The cellular processes affected by SIRT6 depletion were systematically characterized using gene ontology (GO) analysis. Differentially expressed genes most enriched in response to SIRT6 depletion were associated with nucleosome assembly which includes histone variant genes (Fig 4A). Genes encoding histone variants were significantly downregulated (Fig 4B). Changes in gene expression, including HIST2H2AA4, HIST2H2BE, HIST1H2AC, HIST1H1C, and HIST1H2BK, were further validated by RT-PCR. The mRNA levels of these genes were significantly decreased by SIRT6 depletion (Fig 4C).

### SIRT6 depletion elicits DNA damage and G2/M phase arrest

**Fig 5:**
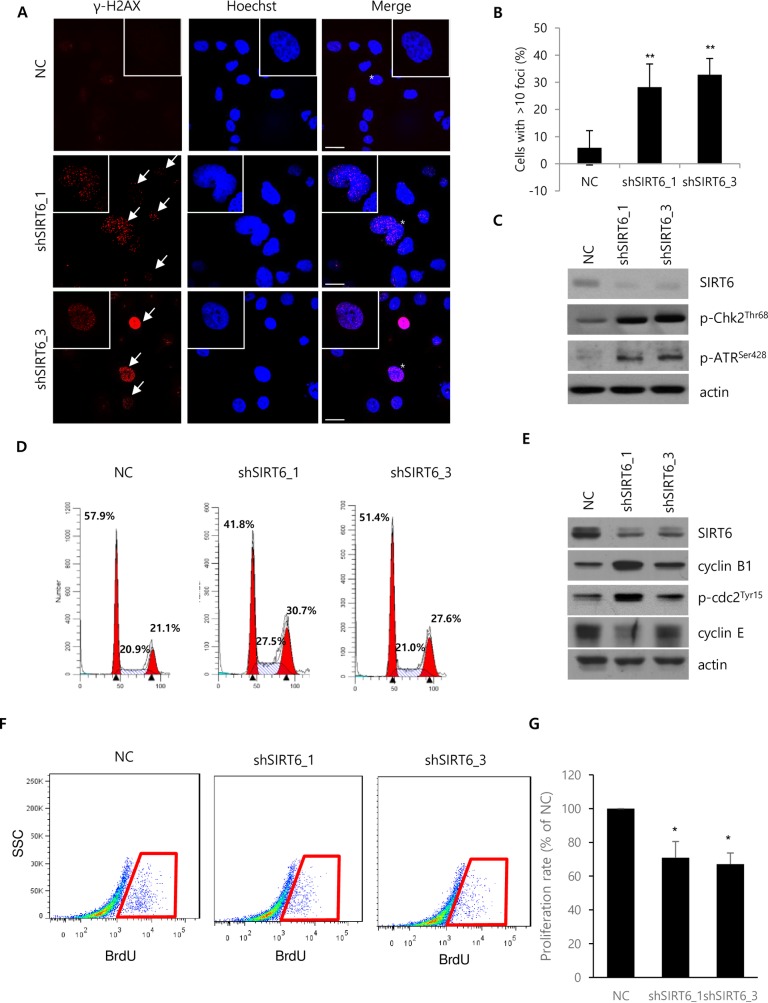
Effect of SIRT6 knockdown on DNA damage and cell cycle progression. (A) Expression of γ-H2AX was assessed by immunocytochemistry using a confocal microscope. (B) Cells with over 10 foci were quantified by counting cells from the randomly obtained images. The quantified cells were compared in the histogram. The scale bar indicates 30 μM. (C) The expression of indicated proteins was assessed by western blotting. Cells for western blotting and immunocytochemistry were harvested or fixed 5 days after viral transduction. (D) Propidium iodide staining was performed to determine the cell cycle distribution in Hep3B cells 5 days after infection with lentiviruses containing NC or shSIRT6. The numbers of Hep3B cells at the G1, S, and G2/M phases were quantified by FACS analysis. The Modifit program was used for data analysis. (E) Expression of cell cycle-related proteins and SIRT6 were assessed by western blotting. Cells for microarray, RT-PCR, and western blotting were harvested 5 days after viral transduction. All data are representative of three independent experiments. (F) Representative FACS analysis of BrdU incorporation. (G) Cell proliferation rate of NC and shSIRT6-depleted cells as quantified by FACS analysis of four independently performed experiments. (**P<0.01, *P<0.05).

DNA damage was confirmed by immunofluorescent staining of γ-H2AX (Fig 5A) because DNA damage was suggested to induce histone loss[24–26]. SIRT6-depleted Hep3B cells had more γ-H2AX-positive foci than control cells (Fig 5A and 5B). The effect of SIRT6 depletion on the expression of DNA damage responsive proteins was investigated by western blotting. The expression of p-chk2^Thr68^ and p-ATR^Ser428^ was increased in SIRT6-depleted Hep3B cells (Fig 5C). Additionally, the mRNA levels of many genes involved in the response to DNA damage were significantly changed by SIRT6 depletion, according to microarray and GO analyses (*P* = 0.073605). Majority of genes involved in the response to DNA damage were significantly upregulated (S1 Fig). Because DNA damage can affect cell cycle progression[27], FACS analysis was performed on SIRT6-depleted Hep3B cells. FACS analysis revealed that more SIRT6-depleted cells were arrested in the G2/M phase of the cell cycle than control cells (Fig 5D). Knockdown of SIRT6 upregulated the expression of cyclin B1 and p-cdc2^Tyr15^, and downregulated the expression of cyclin E (Fig 5E). Since an increase in G2/M phase is also characteristic for proliferating cells, BrdU assay was performed to evaluate the portion of proliferating cells. Flow cytometry analyses revealed that the portion of BrdU-positive cells decreased in SIRT6-depleted Hep3B cells (Fig 5F and 5G).

## Discussion

**Fig 6:**
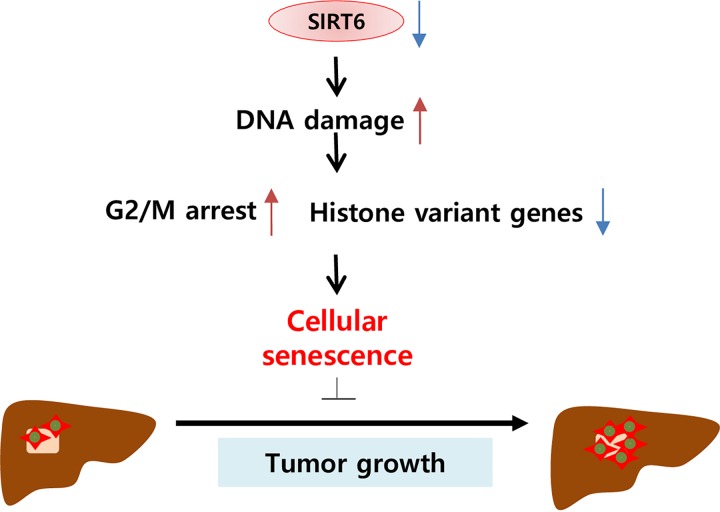
Schematic diagram of SIRT6 depletion in HCC. SIRT6 depletion eventually leads to cellular senescence via the induction of DNA damage, which causes G2/M phase arrest and downregulation of genes encoding histone variants.

In this study, we highlighted the role of SIRT6 which is upregulated in HCC. Mechanistically, SIRT6 silencing by shRNA induced cellular senescence in the p53/p21- and p16/Rb-pathway independent manners. SIRT6 depletion also caused DNA damage, which could promote both the downregulation of genes encoding histone variants associated with nucleosome assembly and cell cycle arrest in the G2/M phase (Fig 6). Our findings demonstrate the oncogenic potential of SIRT6 in HCC and suggest SIRT6 as a potential therapeutic target for HCC.

SIRT6 was upregulated in HCC tissues and its depletion suppressed the growth of HCC, supporting its oncogenic potential. Reports on SIRT6 expression in HCC have been controversial and inconsistent. In contrast with our observations, SIRT6 expression was reported to be lower in HCC tissue than in normal liver tissue based on an analysis of the publically available Oncomine Cancer Microarray database. In this study, SIRT6 expression was reduced in 45% of 53 human HCCs [14]. However, we demonstrated an upregulation of SIRT6 in HCC cell lines, and consistently higher levels of SIRT6 were confirmed in HCC specimens. Our results are supported by recent observations that SIRT6 was upregulated in 101 paired HCC tissues and 60 paired paraffin-embedded sections and that this increased SIRT6 expression was associated with larger tumors and a poorer overall survival rate [15]. We also observed that SIRT6 silencing inhibited colony formation and anchorage-independent HCC cell growth. Furthermore, SIRT6-depleted Hep3B cells produced smaller HCC tumors *in vivo*. Our findings from *in vitro* and *in vivo* studies, together with the analysis of patient specimens, collectively show that SIRT6 has oncogenic potential in HCC.

SIRT6 point mutation was reported to occur in several types of cancers[19]. However, SIRT6 mutations do not seem to naturally and frequently occur in HCC according to the public databases providing SIRT6 status in HCCs, indicating that SIRT6 can be active in most HCC and the effect of SIRT6 depletion is mediated by absence of active SIRT6. Discrepancy between previous reports and data from public databases regarding the status of SIRT6 in HCC might be explained by the fact that SIRT6 mutation seems to occur in tissue-dependent manners. Kugel et al. showed that SIRT6 mutations were found in tumor types such as non-small-cell lung cancer, renal clear cell carcinoma, cervical carcinoma, and melanoma[19], but not in HCC.

SIRT6 depletion suppressed the growth of HCC by promoting cellular senescence and DNA damage in the present study. A previous report has shown that SIRT6 depletion suppressed HCC cell growth and promoted cellular apoptosis by upregulating Bax expression[15]. In agreement with these findings, we also confirmed that some of SIRT6-depleted cells were undergone apoptosis as judged by the Hoechst staining (S2 Fig). Senescence and apoptosis pathways are simultaneously involved in stress responses including DNA damage, and it is the particular wiring of each cell type that decides whether senescence or apoptosis will occur first[28]. In some conditions, apoptosis is the consequence of overwhelming stress, whereas senescence is a response to less severe damage[28]. Indeed, a mixture of apoptosis and senescence was reported in human diploid fibroblasts exposed to cellular stress[29]. Uneven silencing of SIRT6 by shRNA among cells would reflect different degrees of SIRT6 downregulation, which might induce different levels of DNA damage. These differences could explain the different phenotypes, such as apoptosis and senescence, observed in HCC cells.

Acute cellular senescence is one of mechanisms to restrain proliferation of potentially tumorigenic cells[23, 28]. SIRT6 silencing suppressed HCC cell growth by induction of cellular senescence. Consistent with our finding, SIRT6 depletion was reported to induce cellular senescence in several model cell lines. However, most studies on the inhibitory role of SIRT6 in senescence were performed using non-tumorigenic cell lines such as bronchial epithelial cells[30, 31], human fibroblast[32], endothelial cell[33, 34], chondrocyte[35] and keratinocyte[36] in the points of anti-aging effects of SIRT6. In this report, inhibitory roles of SIRT6 in senescence were investigated using tumorigenic cell lines in a normal condition. Though a group recently reported the inhibitory role of SIRT6 in senescence using HCC cell lines, they performed all experiments under the treatment of TGF-1β/H_2_O_2_/HOCI which could induce cellular senescence[37]. In this context, senescence was induced by activating the classical p16 and p21 pathways[37]. However, our results indicated that SIRT6 depletion without the treatment of TGF-1β/H_2_O_2_/HOCI promoted cellular senescence in the p16/Rb- and p53/p21-independent manners. As Hep3B cells do not express p53 and Rb, and p53 is mutated in Huh-7 cells[16], Rb, p53, and p21 expression was not detected in Hep3B cells and p53, p21, p16, and Rb expression was not affected by SIRT6 depletion in Huh-7 cells. Because senescence is frequently associated with DNA damage, the observed senescence could represent a cellular response to DNA damage caused by the loss of SIRT6. In the present study, the loss of SIRT6 increased DNA damage and increased the expression of p-ATR^Ser428^ and p-chk2^Thr68^. The serine/threonine protein kinases ATM and ATR were activated as a part of the genotoxic stress response and phosphorylated chk2, chk1, and H2AX[38]. The induction of DNA damage by SIRT6 silencing could be supported by several studies investigating SIRT6-mediated DNA repair. SIRT6 promotes DNA DSB repair by stabilizing the DNA-PK catalytic subunit (a DSB repair factor) in the chromatin adjacent to DSB[39] or by deacetylating the C-terminal binding protein interacting protein crucial in DSB repair[40]. SIRT6 also contributes to DSB repair by stimulating the poly-ADP-ribosylase activity of poly[ADP-ribose] polymerase 1 in response to DNA damage[41]. Thus, DNA damage may not be repaired properly in SIRT6-depleted cells because SIRT6 promotes DNA repair. Taken together, these findings demonstrated that SIRT6 depletion increased DNA damage, causing subsequent DNA damage responses and eventual cellular senescence in HCC.

In consistent with the previous reports that DNA damage is linked to altered cell cycle distribution[27] and histone loss[25, 26], our expression and cell cycle analyses showed that SIRT6 depletion downregulated histone-encoding genes and promoted altered cell cycle distribution. Change of the chromatin structure by downregulated histone levels could be a cause of cellular senescence[24]. Downregulation of histone levels caused chromatin to be more open, which led to inappropriate access to DNA, disrupting transcriptional regulation and chromatin destabilization[24, 25]. Maintenance of the fundamental chromatin structure was reported to be important for delaying the aging process and overexpression of histones extends life span[42]. Thus, histone loss in SIRT6-deficient cells might accelerate cellular senescence by chromatin destabilization. Usually, cyclin B1 reaches maximum level at G2/M phase and cyclin E does at G1/S transition and its level is relatively low at G2/M phase[43]. The accumulation of p-cdc2^Tyr15^, an inactive form of cdc2, which regulates the G2/M phase transition, could also be an indicator of halted G2/M phase transition[27]. SIRT6 silencing upregulated cyclin B1 and p-cdc2^Tyr15^ expression, and downregulated cyclin E expression. In addition, portion of Brdu-positive cells which were proliferating decreased among SIRT6-depleted cells, indicating that SIRT6-depleted cells were arrested in the G2/M phase. Because G2/M arrest is a common checkpoint mechanism in response to DNA damage[27], it is very likely that the G2/M arrest we observed was initiated by DNA damage caused by SIRT6 depletion. In addition, cancer cell with a defective G1 checkpoint resulted in more profound G2/M arrest upon DNA damage[27] and Hep3B do not express Rb proteins that regulate the G1/S transition[16]. In summary, cellular senescence in SIRT6-depeleted HCC cells was induced by DNA damage, which might downregulate histone-encoding genes associated with nucleosome assembly and promote G2/M arrest.

In summary, we have demonstrated that SIRT6 plays an oncogenic role in HCC. Our findings showed that SIRT6 depletion inhibited tumor growth by inducing cellular senescence via a p16/Rb- and p53/p21- independent pathway. SIRT6 depletion arrested the cell cycle in the G2/M phase and downregulated histone variants associated with nucleosome assembly, which was attributed to DNA damage (Fig 6). Taken together, our findings suggest that SIRT6 acts as a tumor promoter by preventing DNA damage and cellular senescence, indicating that SIRT6 represents a potential therapeutic target for the treatment of HCC.

## Supporting Information

S1 Fig Expression of genes involved in the response to DNA damage in SIRT6-depleted Hep3B cells. (A) The heat map shows the effect of SIRT6 depletion on genes related to the GO term “response to DNA damage stimulus.” “a” and “b” stand for two independent experiments.
(TIF)

S2 Fig Apoptotic morphological changes in SIRT6-depleted HCC cells. Nuclear morphology was confirmed by Hoechst staining using confocal microscopy in (A) Hep3B and (B) Huh-7 cells with NC and shSIRT6. The red arrow indicates apoptotic cells with the characteristics of shrunken and fragmented nuclei. The scale bar indicates 30 μM.
(TIF)

S3 Fig Uncropped blotting data. (PDF)

S1 Table shRNA sequences targeting SIRT6. (PDF)

S2 Table List of antibodies used for western blotting, immunohistochemistry and immunocytochemistry. (PDF)

S3 Table Real-time quantitative PCR primer and probe sequences for HCC patient tumor samples. (PDF)

S4 Table Primer sequences for real-time quantitative PCR for Hep3B cells. (DOCX)
