# Supplementary figures and images for: SIRT6 Depletion Suppresses Tumor Growth by Promoting Cellular Senescence Induced by DNA Damage in HCC
Source: PLoS One. 2016 Nov 8;11(11):e0165835. doi: 10.1371/journal.pone.0165835 (PMC5100879; doi:10.1371/journal.pone.0165835)

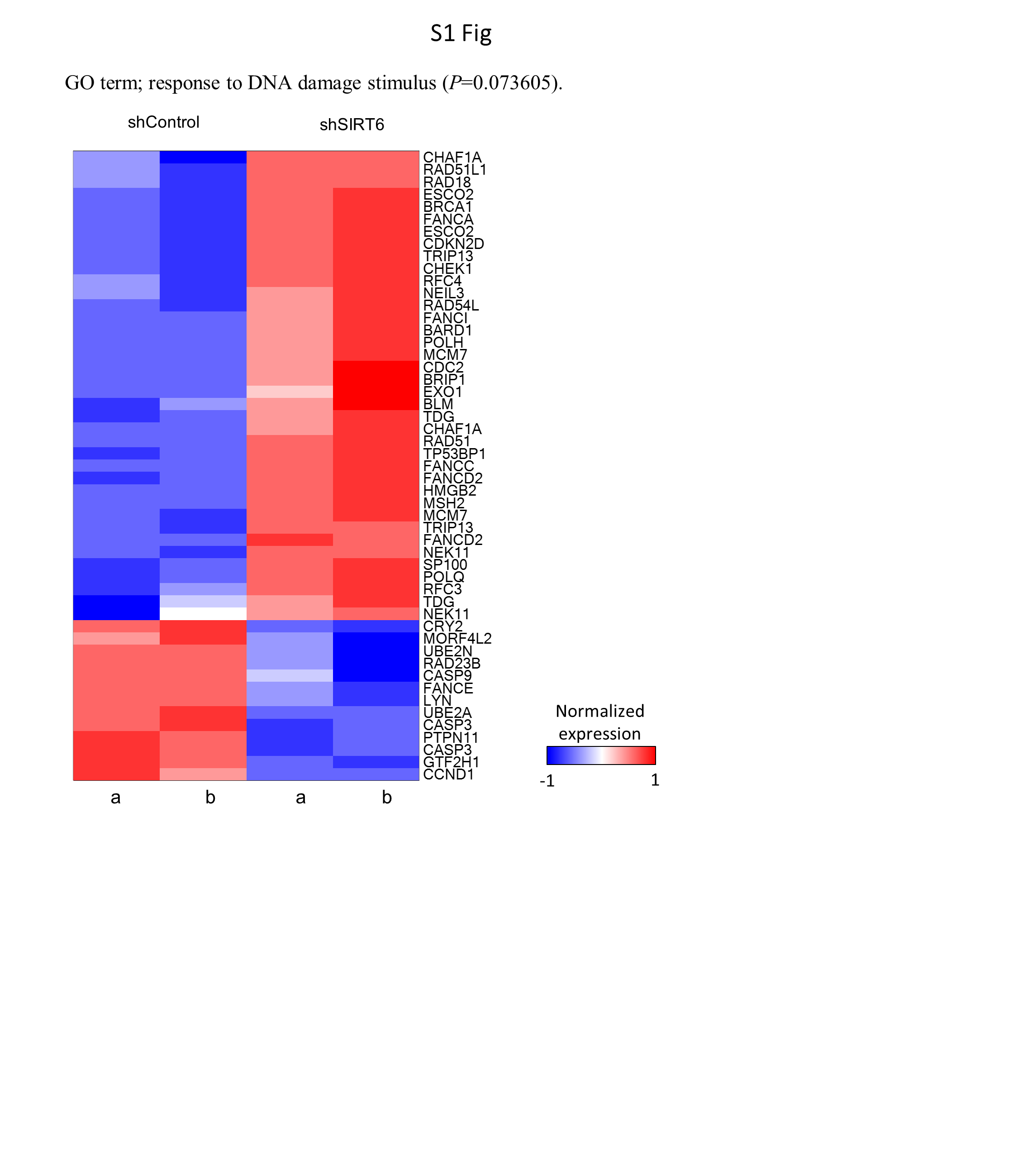

Supplement: S1 Fig — (A) The heat map shows the effect of SIRT6 depletion on genes related to the GO term “response to DNA damage stimulus.” “a” and “b” stand for two independent experiments. (TIF) [file pone.0165835.s001.tif]

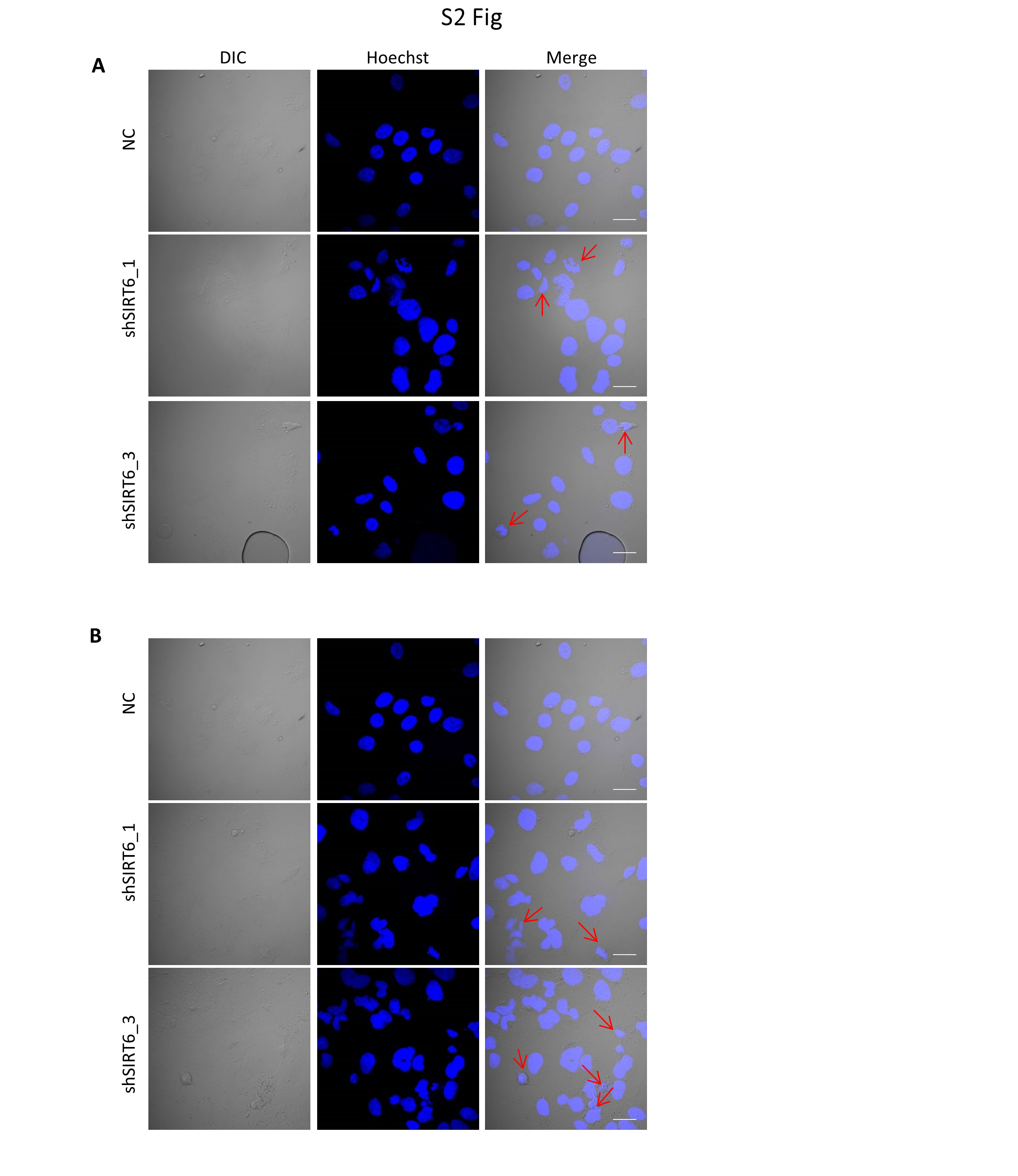

Supplement: S2 Fig — Nuclear morphology was confirmed by Hoechst staining using confocal microscopy in (A) Hep3B and (B) Huh-7 cells with NC and shSIRT6. The red arrow indicates apoptotic cells with the characteristics of shrunken and fragmented nuclei. The scale bar indicates 30 μM. (TIF) [file pone.0165835.s002.tif]

Fig 1

A.

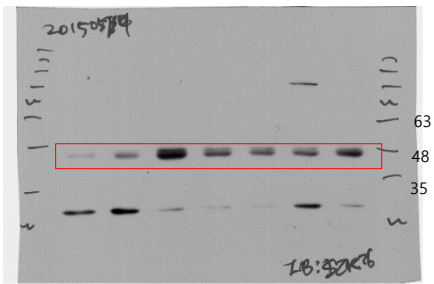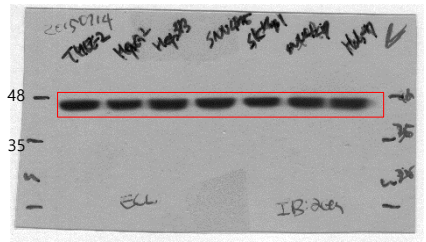

C.

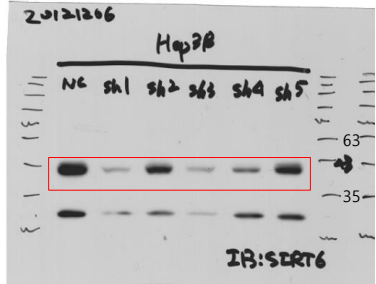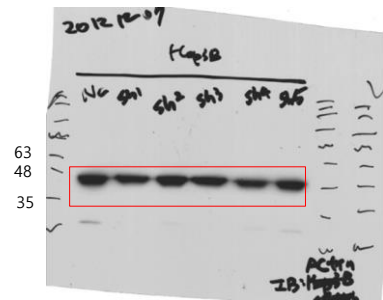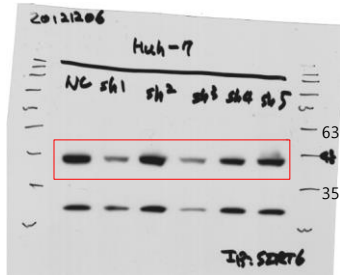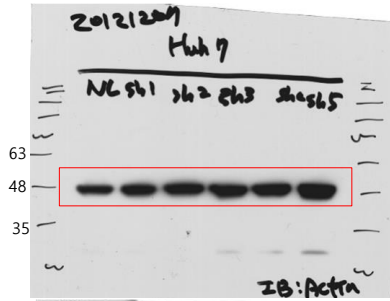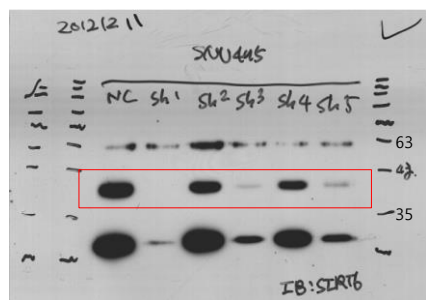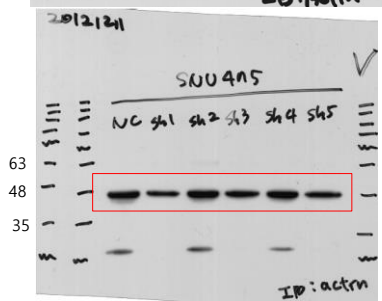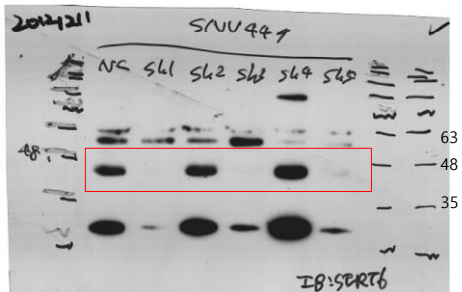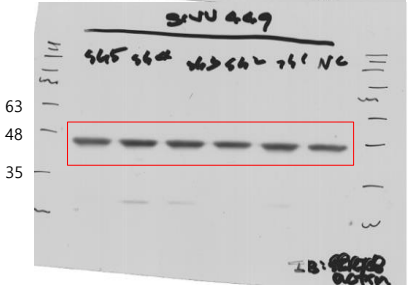

Sup. Fig 3

**Fig 3****D.**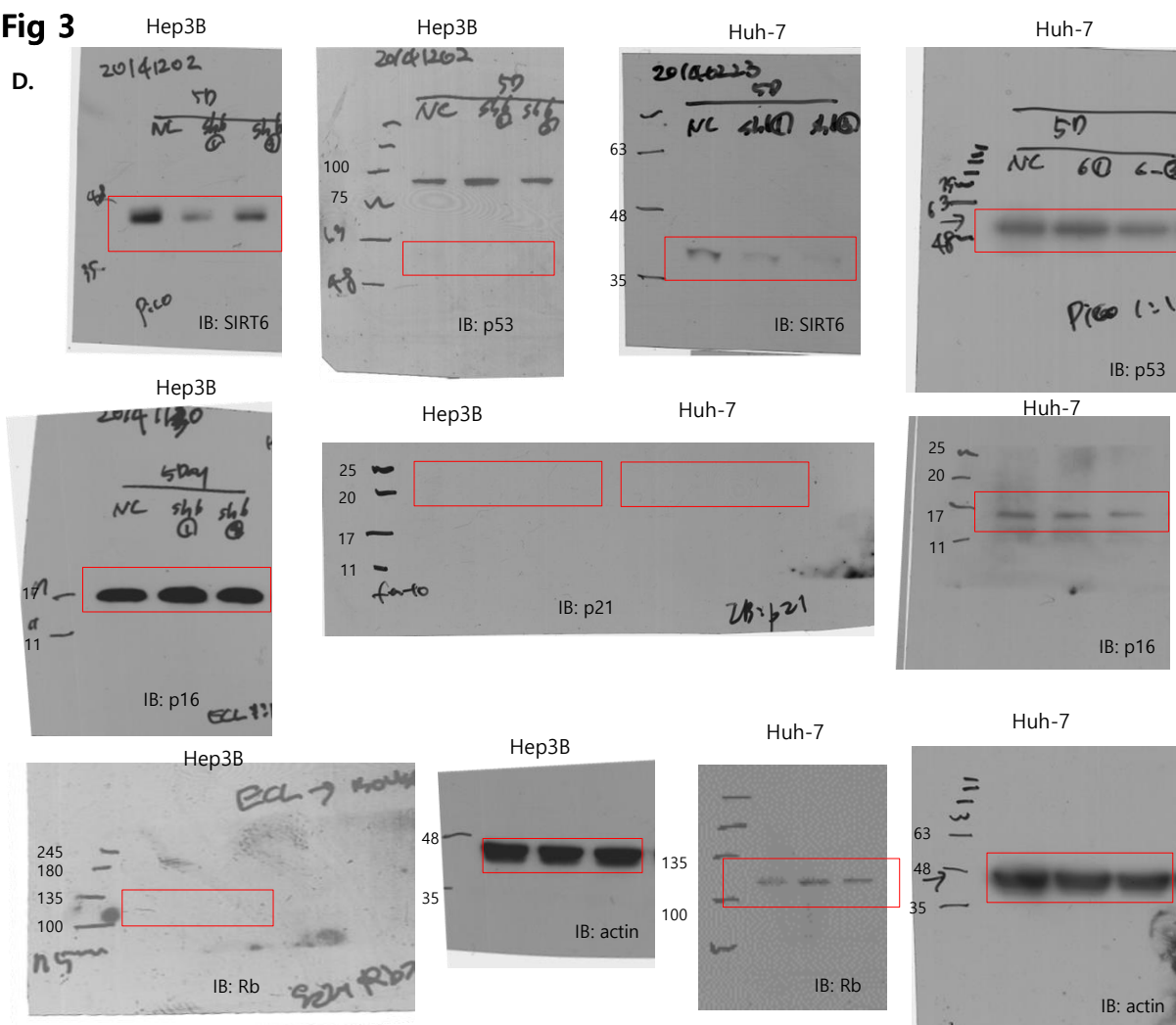**Sup. Fig 3**

Fig 4

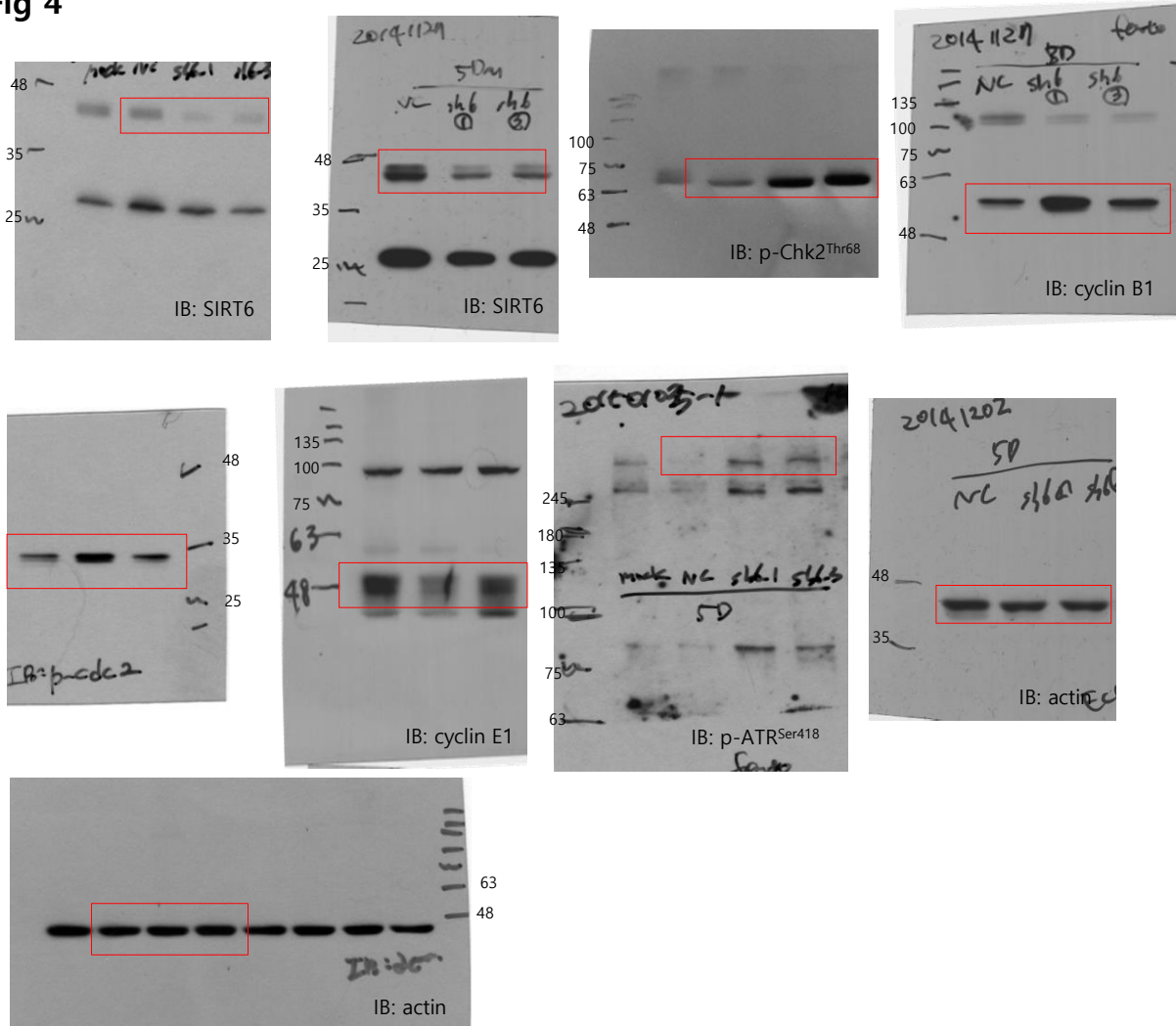

Sup. Fig 3

Supplement: S3 Fig — (PDF) [file pone.0165835.s003.pdf]
