# Supplementary material for: SIRT6 Depletion Suppresses Tumor Growth by Promoting Cellular Senescence Induced by DNA Damage in HCC
Source: PLoS One. 2016 Nov 8;11(11):e0165835. doi: 10.1371/journal.pone.0165835 (PMC5100879; doi:10.1371/journal.pone.0165835)
Supplement: S1 Table — (PDF) [file pone.0165835.s004.pdf]

S1 Table. shRNA sequences targeting SIRT6

C1	TRCN0000050473, CCGGTGGAAGAATGTGCCAAGTGTACTCGAGTACACTTGGCACATTCTTCCATTTTTG
C2	TRCN0000050474, CCGGGCAGTCTTCCAGTGTGGTGTTCTCGAGAACACCACACTGGAAGACTGCTTTTTG
C3	TRCN0000050475, CCGGGCTGGGTACATCGCTGCAGATCTCGAGATCTGCAGCGATGTACCCAGCTTTTTG
C4	TRCN0000050476, CCGGCACCCGGATCAACGGCTCTATCTCGAGATAGAGCCGTTGATCCGGGTGTTTTTG
C5	TRCN0000050477, CCGGCACGGGAACATGTTTGTGGAACTCGAGTTCCACAAACATGTTCCCGTGTTTTTG
