# Supplementary material for: SIRT6 Depletion Suppresses Tumor Growth by Promoting Cellular Senescence Induced by DNA Damage in HCC
Source: PLoS One. 2016 Nov 8;11(11):e0165835. doi: 10.1371/journal.pone.0165835 (PMC5100879; doi:10.1371/journal.pone.0165835)
Supplement: S2 Table — (PDF) [file pone.0165835.s005.pdf]

Name of antibody	monoclonal or polyclonal	host species	supplier	catalog number	antigens	final dilution	reference
SIRT6	polyclonal	rabbit	Bethyl	A302-452A	residues 305 and 355 of human sirtuin 6	1:1000	Lee et al, Proteomics, 2014 Jul; 14(13-14):1610-22
actin	polyclonal	goat	Sata Cruz	sc1615	C-terminus of actin of human	1:400	Lee et al, Proteomics, 2014 Jul; 14(13-14):1610-22
Ki67	monoclonal	mouse	Dako	M7240	human recombinant peptide corresponding to a 1002 bp Ki67-cDNA fragment	1:200	Lee et al, Oncotarget, 2015 Oct 6;6(30):30130-48
p53	monoclonal	mouse	Sata Cruz	sc126	N-terminal epitope mapping between amino acid residues 11-25 of p53 of human origin	1:400	Lee et al, Oncotarget, 2015 Oct 6;6(30):30130-48
p21	monoclonal	mouse	Sata Cruz	sc6246	amino acid 1-159 representing full length p21 of mouse origin	1:200	Lee et al, Oncotarget, 2015 Oct 6;6(30):30130-48
Rb	monoclonal	mouse	Cell Signaling	9309	Rb-C fusion protein containing residues 701-928 of human Rb	1:1000	Lee et al, Oncotarget, 2015 Oct 6;6(30):30130-48
p16	monoclonal	mouse	Sata Cruz	sc56330	full length recombinant p16 of human origin	1:200	Lee et al, Oncotarget, 2015 Oct 6;6(30):30130-48
p-Chk2^{Thr68}	monoclonal	rabbit	Cell Signaling	2197	synthetic peptide coresponding to residues surrounding Thr68 of Chk2	1:1000	Kwon et al, Int J Cancer 2013 Feb 15; 132(4):832-42
p-ATR^{Ser428}	polyclonal	rabbit	Cell Signaling	2853	synthetic peptide coresponding to residues surrounding Ser428 of ATR	1:1000	TL et al., Cell Rep, 2016 Apr 13. pii: S2211-1247(16)30367-9
p-cdc2^{Tyr15}	polyclonal	rabbit	Cell Signaling	9111	synthetic peptide coresponding to residues surrounding Tyr15 of cdc2	1:1000	Kim et al., PLoS One, 2016 Mar 30; 11 (3):e0152591
cyclin B1	polyclonal	rabbit	Cell Signaling	4138	residues near the amino terminus of human cyclin B1	1:1000	Lee et al, Oncotarget, 2015 Oct 6;6(30):30130-48
cyclin E1	polyclonal	mouse	Cell Signaling	4129	recombinant human cyclin E1	1:1000	Lee et al, Oncotarget, 2015 Oct 6;6(30):30130-48
γH2AX	monoclonal	rabbit	Cell Signaling	2577	synthetic phosphopeptide coresponding to residues surrounding Ser139 of human H2AX	1:200	Kwon et al, Int J Cancer 2013 Feb 15; 132(4):832-42
