# Supplementary material for: SIRT6 Depletion Suppresses Tumor Growth by Promoting Cellular Senescence Induced by DNA Damage in HCC
Source: PLoS One. 2016 Nov 8;11(11):e0165835. doi: 10.1371/journal.pone.0165835 (PMC5100879; doi:10.1371/journal.pone.0165835)
Supplement: S3 Table — (PDF) [file pone.0165835.s006.pdf]

S3 Table. Real-time quantitative PCR primer and probe sequences for HCC patient tumor samples

Genes		Sequences
B2M	F	CATTCGGGCCGAGATGTCT
	R	CTCCAGGCCAGAAAGAGAGAGTAG
	P	CCGTGGCCTTAGCTGTGCTCGC
GAPDH	F	CACATGGCCTCCAAGGAGTAA
	R	TGAGGGTCTCTCTCTTCCTCTTGT
	P	CTGGACCACCAGCCCCAGCAAG
HMBS	F	CCAGGGATTTGCCTCACCTT
	R	AAAGAGATGAAGCCCCCACAT
	P	CCTTGATGACTGCCTTGCCTCCTCAG
HPRT1	F	GCTCGAGATGTGATGAAGGAGAT
	R	CCAGCAGGTCAGCAAAGAATT
	P	CCATCACATTGTAGCCCTCTGTGTGCTC
SDHA	F	CACCTAGTGGCTGGGAGCTT
	R	GCCCAGTTTTATCATCTCACAAGA
	P	TGGCACTTACCTTTGTCCCTTGCTTCA
SIRT6	F	TTCCTGGTCAGCCAGAACGT
	R	GGCACATTCTTCCACAAACATG
	P	CCCCAGGGACAAACTGGCAGAGCT
