# Supplementary material for: SIRT6 Depletion Suppresses Tumor Growth by Promoting Cellular Senescence Induced by DNA Damage in HCC
Source: PLoS One. 2016 Nov 8;11(11):e0165835. doi: 10.1371/journal.pone.0165835 (PMC5100879; doi:10.1371/journal.pone.0165835)
Supplement: S4 Table — (DOCX) [file pone.0165835.s007.docx]

**S4 Table. Primer sequences for real-time quantitative PCR in Hep3B cells**

| **Genes** |  | **Sequences** |
| --- | --- | --- |
| HIST2H2AA3 | F  R | CGACGAGGAACTGAACAAGC  CTTTTGAGTTCACAGGTGCCC |
| HIST2H2BE | F  R | GAAGGGTGCTGTTCATTTCC  ACTGAGAAGCATAAGGGTGG |
| HIST1H2AC | F  R | GACAACAAGAAGACTCGCATC  GGAGCTCAGATACCTGTCAAA |
| HIST1H1C | F  R | TAAAGAGCGTAGCGGAGTTT  GCTGCCTTCTTGTTGAGTTT |
| HIST1H2BK | F  R | ACATCTTCGAACGCATCGC  GAGAAAGTCCCTCCTTGGC |
| IL8 | F  R | AGCACACAAGCTTCTAGGAC  TAGCACTCCTTGGCAAAACT |
| CXCL1 | F  R | CAATCCTGCATCCCCCATAG  CATTAGGCACAATCCAGGTG |
| CXCL2 | F  R | ACCCAAGTTAGTTCAATCCTG  ACATACATTTCCCTGCCGTC |
| CXCL3 | F  R | AGTGTGAATGTAAGGTCCCC  GGAAGTGTCAATGATACGCTG |
| GAPDH | F  R | CCTGGTATGACAACGAATTTGGC  GTACATGACAAGGTGCGGCTC |
